# Sulfated glycosaminoglycans and low-density lipoprotein receptor mediate the cellular entry of *Clostridium novyi* alpha-toxin

**DOI:** 10.1038/s41422-021-00510-z

**Published:** 2021-05-10

**Authors:** Yao Zhou, Diyin Li, Jianhua Luo, Aizhong Chen, Xingxing Li, Zhenrui Pan, Li Wan, Liuqing He, Danyang Li, Yanyan Li, Min Dong, Liang Tao

**Affiliations:** 1 Key Laboratory of Structural Biology of Zhejiang Province, School of Life Sciences, Westlake University, Hangzhou, Zhejiang China; 2 Center for Infectious Disease Research, Westlake Laboratory of Life Sciences and Biomedicine, Hangzhou, Zhejiang China; 3 Institute of Basic Medical Sciences, Westlake Institute for Advanced Study, Hangzhou, Zhejiang China; 4 Department of Urology, Boston Children’s Hospital, Boston, MA USA; 5 Department of Surgery and Department of Microbiology, Harvard Medical School, Boston, MA USA

**Keywords:** Mechanisms of disease, Molecular biology

Dear Editor,

*Clostridium novyi* (*C. novyi*) is a spore-forming anaerobic bacterium and opportunistic pathogen causing severe infectious diseases in humans and animals including gas gangrene, myositis, necrotic hepatitis, and sepsis.^1,2^ The alpha-toxin (Tcnα) is the major virulence factor of *C. novyi* and is produced by all known pathogenic *C. novyi* types.^1^ Tcnα belongs to the large clostridial toxin (LCT) family which also includes *Clostridioides difficile* toxin A (TcdA) and toxin B (TcdB), *Paeniclostridium sordellii* lethal toxin (TcsL) and hemorrhagic toxin (TcsH), and *Clostridium perfringens* large cytotoxin (TpeL). Like other LCTs, Tcnα binds to the surface of the target cell and enters the cell via receptor-mediated endocytosis. The enzymatic domain is then delivered into the cytosol and glucosylates small Rho-family GTPases using UDP-N-acetylglucosamine as a co-substrate, leading to cytoskeleton disruption and cell death.^3^ Recently, the cellular receptors of several LCTs, including TpeL, TcdB, TcdA, and TcsL have been identified.^4–10^ Tcnα and TcsH are the only two major members of the LCT family with their host receptors remaining unclear.

To identify potential receptors for Tcnα, we conducted genome-wide CRISPR/Cas9-mediated knockout (KO) screens. HeLa cells stably expressing Cas9 (parental cell line, referred to as the wildtype (WT) thereafter) were transduced with a lentiviral single guide RNA (sgRNA) library (GeCKO v2) and subjected to three rounds of selection with Tcnα (Fig. 1a). Next-generation sequencing of sgRNAs from the surviving cells showed that four of the top five enriched genes, including *B4GALT7, B3GALT6, EXT1*, and *SLC35B2*, encode enzymes involved in heparan sulfate biosynthesis. Several other genes encoding enzymes in the heparan sulfate biosynthesis pathways, such as *B3GAT3*, *EXT2*, *HS6ST3*, and *EXTL3* were also greatly enriched (>10-fold) from our screens (Fig. 1b; Supplementary information, Table S1).

**Fig. 1:**
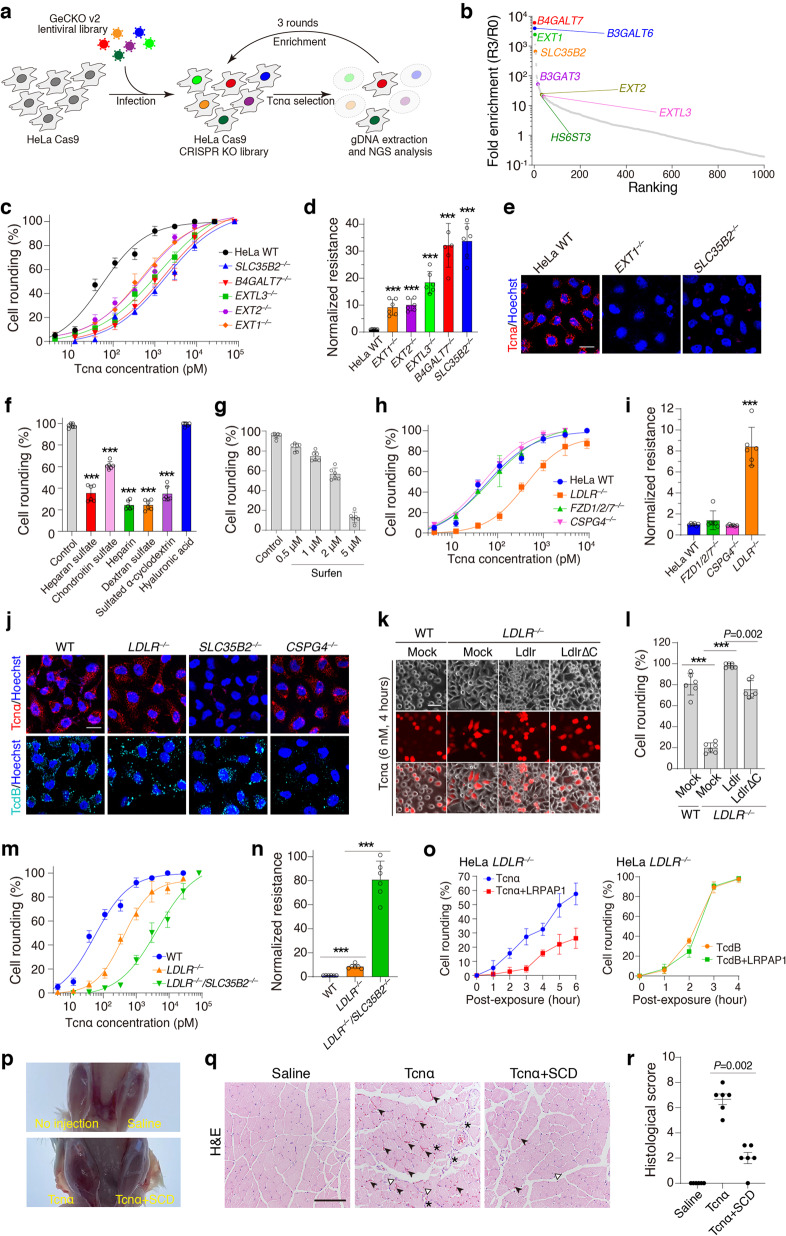
Sulfated glycosaminoglycans and LDLR mediate the cellular entry of Tcnα. **a** Schematic of the screening process using Tcnα on HeLa cells with GeCKO v2 library. **b** Genes identified are ranked and plotted based on fold-enrichment of their gRNA from the beginning (R0) to 3-round post-toxin selection (R3). Genes involved in heparan sulfate biosynthesis are marked. **c** Sensitivities of the WT, *SLC35B2*^*‒/‒*^, *B4GALT7*^*‒/‒*^*, EXTL3*^*‒/‒*^*, EXT1*^*‒/‒*^, and *EXT2*^*‒/‒*^ HeLa cells to Tcnα were measured using the cytopathic cell-rounding assay. The percentages of rounded cells were quantified, plotted, and fitted. **d** Resistance of *SLC35B2*^*‒/‒*^*, B4GALT7*^*‒/‒*^*, EXTL3*^*‒/‒*^*, EXT1*^*‒/‒*^, and *EXT2*^*‒/‒*^ HeLa cells to Tcnα were normalized to the level of WT cells based on CR_50_. (***P < 0.001 vs WT). **e** Confocal fluorescence images of Rhodamine-labeled Tcnα binding to the WT, *EXT1*^*‒/‒*^, and *SLC35B2*^*‒/‒*^ HeLa cells. Cell nuclei were labeled with Hoechst. **f** HeLa cells were exposed to either Tcnα (7.5 nM) or Tcnα pre-mixed with the indicated glycans (1 mg/mL). The levels of cell rounding with 6 h incubation were plotted in a bar chart. (***P < 0.001 vs control). **g** HeLa cells were pre-incubated with surfen of the indicated concentrations and then exposed to Tcnα (10 nM). The levels of cell rounding after 7 h incubation were plotted in a bar chart. **h** Sensitivities of the WT, *LDLR*^*‒/‒*^*, FZD1/2/7*^*‒/‒*^, and *CSPG4*^*‒/‒*^ HeLa cells to Tcnα were measured using the cytopathic cell-rounding assay. The percentages of rounded cells were quantified, plotted, and fitted. **i** Resistance of *LDLR*^*‒/‒*^*, FZD1/2/7*^*‒/‒*^, and *CSPG4*^*‒/‒*^ HeLa cells to Tcnα were normalized to the level of WT cells. (***P < 0.001 vs WT). **j** Confocal fluorescence microscopy showing different binding of Rhodamine-labeled Tcnα or TcdB to the WT, *LDLR*^*‒/‒*^, *SLC35B2*^*‒/‒*^, and *CSPG4*^*‒/‒*^ HeLa cells. Cell nuclei were labeled with Hoechst. **k** Expression of mouse Ldlr restored Tcnα entry into HeLa *LDLR*^*‒/‒*^ cells, resulting in cell rounding; expression of mouse Ldlr∆C also restored Tcnα entry but less efficiently. Red fluorescence (mCherry) marked transfected cells. **l** Cell rounding among fluorescence positive cells shown in **k** was quantified and plotted in a bar chart. (***P < 0.001). **m** Sensitivities of the HeLa WT, *LDLR*^*‒/‒*^, and *LDLR*^*‒/‒*^*/SLC35B2*^*‒/‒*^ cells to Tcnα were measured using the cytopathic cell-rounding assay. The percentages of rounded cells were quantified, plotted, and fitted. **n** Resistance of *LDLR*^*‒/‒*^, and *LDLR*^*‒/‒*^*/SLC35B2*^*‒/‒*^ HeLa cells to Tcnα were normalized to the level of WT cells. (***P < 0.005). **o** LRPAP1 in culture medium further protected HeLa *LDLR*^*‒/‒*^ cells from Tcnα (left panel, 5 nM) but not TcdB (right panel, 2.5 pM) under the molar ratio of 1:1000 (toxin vs LRPAP1). The percentages of rounded cells were quantified and plotted over time. **p** Anatomy of the mouse tibialis anterior muscles after i.m. injection of saline, Tcnα, or Tcnα + SCD. **q** Representative images of H&E staining sections from the mice tibialis anterior muscles after i.m. injection of saline, Tcnα alone, or Tcnα + SCD. Asterisks indicate inflammatory cell infiltrate, solid arrowheads indicate hemorrhage, and hollow arrowheads indicate central nuclei. **r** Histopathological scores were accessed and summarized based on hemorrhage, inflammatory cell infiltration, and muscle fiber injury. Data are means ± SD, *n* = 6 in **c**, **d**, **f**–**i**, and **l**–**o**, two-sided Student’s *t*-test. Data are means ± SEM, *n* = 6 in **r**, Mann–Whitney test. Scale bar represents 50 μm in **e**, **j**, and **k**, and 100 μm in **q**.

To validate the role of heparan sulfate in the cellular binding/entry of Tcnα, we generated the HeLa *EXT1*^*‒/‒*^, *EXT2*^*‒/‒*^, *EXTL3*^*‒/‒*^, *B4GALT7*^*‒/‒*^, and *SLC35B2*^*‒/‒*^ cells via CRISPR/Cas9-mediated KO. The cytopathic cell rounding experiment (toxin concentration that results in 50% cell rounding is defined as CR_50_) showed that all these KO cells exhibited increased resistance (~10 to 30-fold based on CR_50_) against Tcnα compared to the WT cells (Fig. 1c, d). Heparan sulfate is usually anchored to core proteins as heparan sulfate proteoglycans (Supplementary information, Fig. S1) and is abundant on various cell types, thus may serve as universal cell surface attachment factors for Tcnα. We labeled Tcnα with fluorescent dye Rhodamine and then examined its surface binding to the HeLa cells. Rhodamine-labeled Tcnα showed similar toxicity as Tcnα (Supplementary information, Fig. S2). As expected, both *EXT1*^*‒/‒*^ and *SLC35B2*^*‒/‒*^ HeLa cells showed drastically reduced surface binding of Tcnα when compared with the WT cells (Fig. 1e).

Among the above tested KO cells, HeLa *B4GALT7*^*‒/‒*^ and *SLC35B2*^*‒/‒*^ cells showed particularly high resistance (>30-fold) against Tcnα (Fig. 1d). Unlike EXT1, EXT2, and EXTL3, which only catalyze the heparan sulfate biosynthesis, B4GALT7 and SLC35B2 are involved in the biosynthesis of various sulfated glycans. B4GALT7 catalyzes the initiation of glycosaminoglycan side chain synthesis and SLC35B2 transports adenosine-3′-phospho-5′-phosphosulfate (PAPS), a sulfate donor, from the cytosol into the Golgi (Supplementary information, Fig. S1). To examine whether other sulfated glycosaminoglycans could also be recognized by Tcnα, we performed competition assays using a panel of glycans structurally related to heparan sulfate. Pre-incubation of Tcnα with heparan sulfate, chondroitin sulfate, heparin, dextran sulfate, and sulfated α-cyclodextrin (SCD) all reduced level of cell rounding, whereas hyaluronic acid, the non-sulfated glycosaminoglycan, had no inhibitory effect (Fig. 1f; Supplementary information, Fig. S3a). Consistently, pre-treatment of cells with surfen (bis-2-methyl-4-amino-quinolyl-6-carbamide), a small molecule that neutralizes the negative charge of sulfated glycosaminoglycans, protected HeLa cells from Tcnα (Fig. 1g; Supplementary information, Fig. S3b). These results demonstrated that Tcnα recognizes sulfated glycans and the negatively charged sulfation group is critical for the interactions.

Synergistic actions between glycosaminoglycans and low-density lipoprotein receptor (LDLR) are common for cellular internalization of ligands. To demonstrate whether Tcnα hijacks this pathway for toxin entry, we examined the sensitivity of HeLa *LDLR*^*‒/‒*^ cells to Tcnα using the cell rounding assay. We also included the HeLa *CSPG4*^*‒/‒*^ and *FZD1/2/7*^*‒/‒*^ cells, which are resistant to TcdB,^5^ for comparison. *LDLR*^*‒/‒*^ cells, but not *CSPG4*^*‒/‒*^ and *FZD1/2/7*^*‒/‒*^ cells, showed modestly reduced sensitivity (~8-fold) to Tcnα (Fig. 1h, i). On the other hand, surface binding of Tcnα was diminished in *SLC35B2*^*‒/‒*^ cells but not *LDLR*^*‒/‒*^ and *CSPG4*^*‒/‒*^ cells (Fig. 1j), indicating that sulfated glycosaminoglycans but not LDLR mainly contribute to the surface attachment of Tcnα. LDLR is rapidly and constitutively internalized and recycled between cell membranes and endosomes, and its cytosolic domain is critical for mediating these processes.^11^ We showed that the susceptibility of *LDLR*^*‒/‒*^ cells can be restored by transient transfection of full-length mouse Ldlr (Fig. 1k). Interestingly, transient transfection of an Ldlr mutant lacking the cytosolic domain (residues 1‒824, Ldlr∆C) also restored the susceptibility of *LDLR*^*‒/‒*^ cells but less efficiently (Fig. 1k, l), demonstrating that the fast-recycling ability of LDLR promotes the intoxication of Tcnα in HeLa cells but is not essential. We could not detect obvious binding of Tcnα to LDLR in vitro (Supplementary information, Fig. S4), indicating that either the interaction between Tcnα and LDLR is weak or additional co-factors are needed.

We next examined the sensitivity of the HeLa *LDLR*^*‒/‒*^*/SLC35B2*^*‒/‒*^ cells to Tcnα. These double KO cells showed further increased resistance to Tcnα compared to the LDLR KO cells (Fig. 1m, n), implying the existence of other endocytic portals besides LDLR. LDLR belongs to the LDLR family, of which members often act as redundant receptors for many ligands due to the similarity of their structural domains. Low-density lipoprotein receptor-related protein-associated protein 1 (LRPAP1) binds tightly to most LDLR family members and inhibits their ligand binding abilities. The addition of LRPAP1 at the molar ratio of 1:1000 (toxin vs LRPAP1) further reduced the sensitivity of *LDLR*^*‒/‒*^ cells to Tcnα but not to TcdB (Fig. 1o, Supplementary information, Fig. S5), indicating that other LDLR family members likely serve as redundant endocytic receptors for Tcnα.

Recent studies showed that TcdA uses sulfated glycosaminoglycans as the cell surface attachment factors and LDLR family proteins as the entry receptors.^8,12^ The primary sequences between Tcnα and TcdA are divergent, with only ~30% identity. In the LCT family, TcdA is most similar to TcsH (~78% identity). We demonstrated that TcsH is equally toxic to the WT, *LDLR*^*‒/‒*^, and *SLC35B2*^*‒/‒*^ cells (Supplementary information, Fig. S6a). We further tested the heparin-binding of major LCTs including TcdB, TcsL, TcsH, and Tcnα, only Tcnα bound to the heparin-beads (Supplementary information, Fig. S6b).

Sulfated glycosaminoglycans are widely distributed in soft tissues including muscles, which are major pathologically relevant targets of Tcnα. In vitro binding experiment showed that Rhodamine-labeled Tcnα robustly bound to the tissue sections of mouse skeleton muscles. The toxin binding was drastically reduced when co-incubated with heparin or SCD, but not hyaluronic acid (Supplementary information, Fig. S7), suggesting that sulfated glycosaminoglycans are the major attachment factors mediating Tcnα binding to the muscle fibroblast cells.

Finally, we investigated the role of sulfated glycosaminoglycans in Tcnα-induced myopathogenesis in vivo using a toxin intramuscular (i.m.) injection model in mice. In brief, saline, Tcnα, or Tcnα premixed with SCD was injected into the mouse tibialis anterior muscles. Sulfated cyclodextrins had minimal cytotoxicity and hemolytic activity and are considered as potential ingredients in drug development and pharmaceutical applications.^13^ Therefore, we chose SCD as the inhibitor for the toxin protection assay in vivo. The tibialis anterior muscles injected with Tcnα were visually swollen and congested (Fig. 1p). Hematoxylin and eosin (H&E) staining showed that i.m. injection of Tcnα resulted in severe hemorrhage and inflammatory cell infiltration. Central nuclei, the morphological marker for myopathies such as early necrosis, were also observed (Fig. 1q, r; Supplementary information, Fig. S8). The muscles co-injected with Tcnα and SCD were less swollen and congested than those injected with Tcnα alone (Fig. 1p). Histological analysis further revealed that co-injection of SCD drastically reduced Tcnα-induced hemorrhage, inflammatory cell infiltration, and central nuclei (Fig. 1q, r; Supplementary information, Fig. S8).

In summary, we identified sulfated glycosaminoglycans as key attachment factors for Tcnα via CRISPR/Cas9-mediated KO screens. Sulfated glycosaminoglycans are ubiquitously present on the cell surface thus serve as ideal landing pads for various infectious agents including SARS-COV-2, the emerging virus that causes global pandemics.^14,15^ We further revealed that the synergistic actions between sulfated glycosaminoglycans and LDLR family members facilitate the entry of Tcnα. Finally, we demonstrated that sulfated glycosaminoglycans are key attachment factors responsible for Tcnα-induced myopathies in vivo, and SCD effectively reduced the tissue damage caused by Tcnα in the mouse model. These findings suggest that blocking the interactions between Tcnα and sulfated glycosaminoglycans could be a novel strategy for developing potential therapeutics against Tcnα-induced diseases.

## Supplementary information

Supplementary Information

Supplementary information, Table S1
