# Supplementary material for: Sulfated glycosaminoglycans and low-density lipoprotein receptor mediate the cellular entry of Clostridium novyi alpha-toxin
Source: Cell Res. 2021 May 10;31(8):935–8. doi: 10.1038/s41422-021-00510-z (PMC8107810; doi:10.1038/s41422-021-00510-z)
Supplement: Supplementary file 1 — Supplementary Information [file 41422_2021_510_MOESM1_ESM.pdf]

Supplementary information for "Sulfated glycosaminoglycans and low-density lipoprotein receptor mediate the cellular entry of *Clostridium novyi* alpha toxin"

Supplementary information, Fig. S1

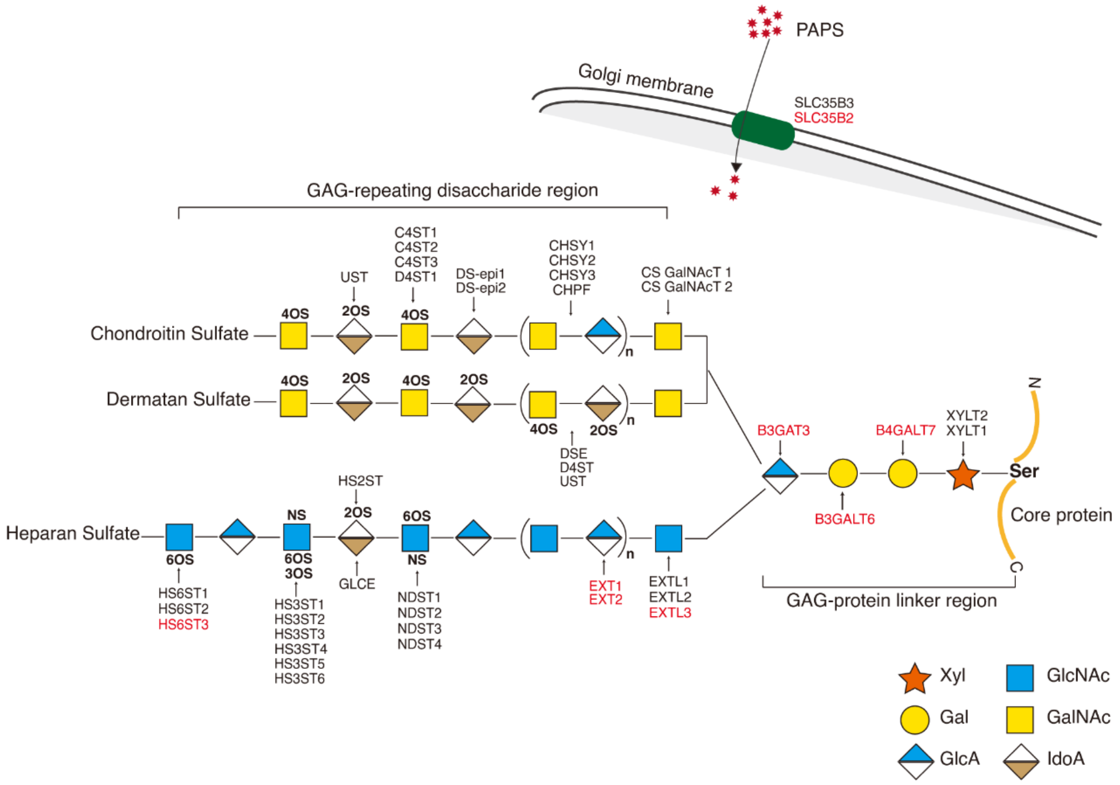

Schematic drawing of biosynthesis pathways of sulfated glycosaminoglycans. Schematic illustration shows the biosynthesis pathways of heparan sulfate, chondroitin sulfate, and dermatan sulfate. Candidates identified in the screen with more than 10-fold enrichment at R3 are marked in red.

Supplementary information, Fig. S2

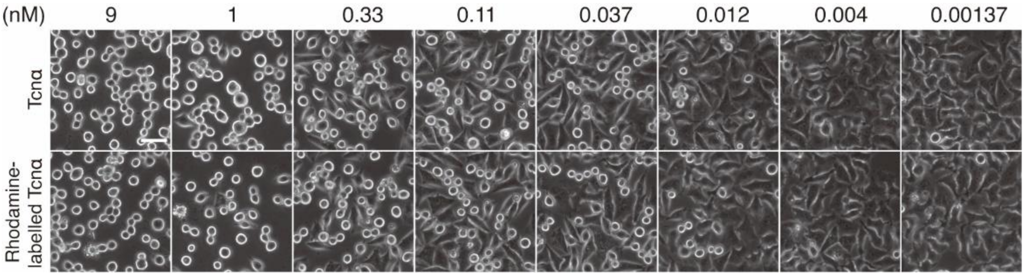

Rhodamine-labeling did not affect the toxicity of Tcnα. HeLa cells were exposed to a serial dilution of Tcnα or Rhodamine-labeled Tcnα as indicated for 10 hours. Tcnα and Rhodamine-labeled induced the same levels of cell rounding. Scale bar represents 50 μm.

Supplementary information, Fig. S3

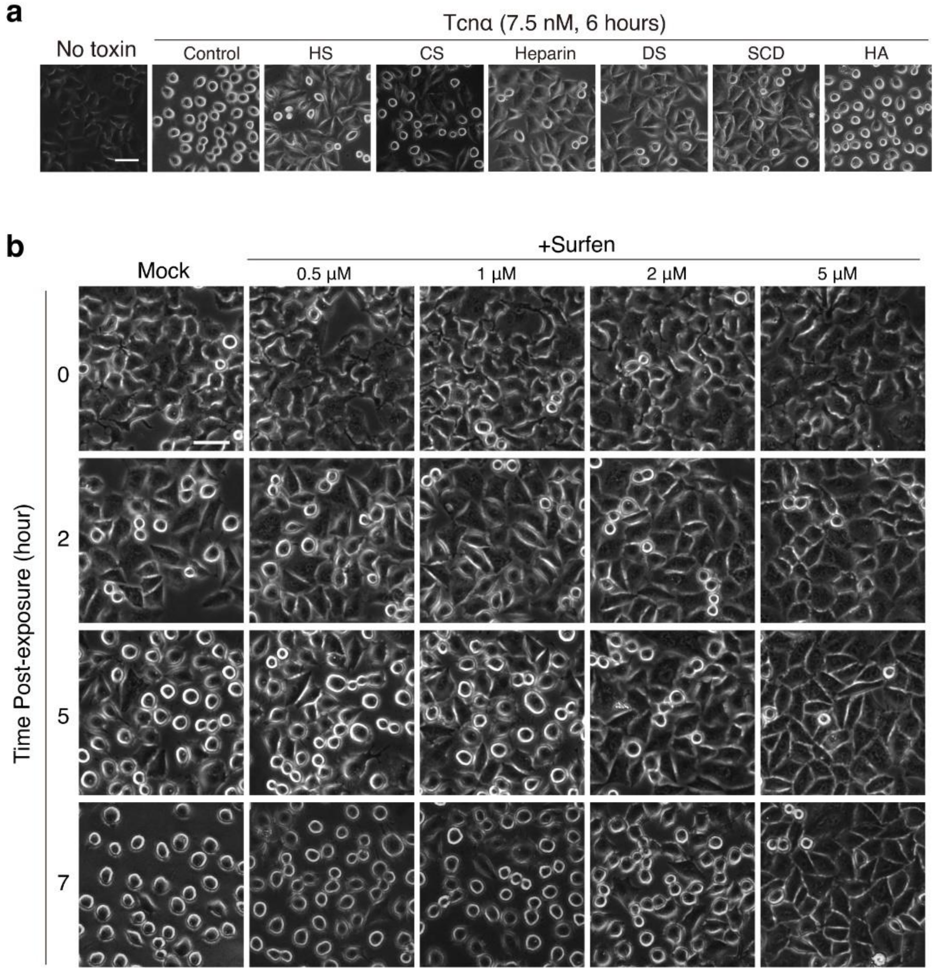

Sulfated glycans and Surfen protect cells from Tcnα. a Representative images showing that pre-incubation of heparan sulfate (HS), chondroitin sulfate (CS), heparin, dextran sulfate (DS), or sulfated α-cyclodextrin (SCD), but not hyaluronic acid (HA) (all at 1 mg/ml), reduced cytopathic effect caused by Tcnα. **b** Representative images showing that pre-incubation of Surfen reduced cytopathic effect caused by Tcnα in a dose-dependent manner. (Scale bar represents 50 μm)

Supplementary information, Fig. S4

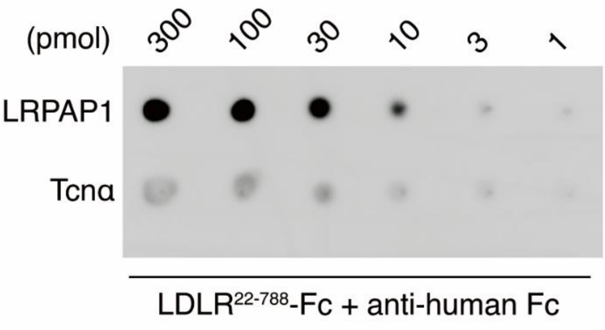

Dot blot assay showed no obvious binding of LDLR$^{22\text{-}788}$-Fc to Tcnα. The indicated amounts of Tcnα or LRPAP1 were immobilized onto nitrocellulose (NC) membrane and blotted with LDLR$^{22\text{-}788}$-Fc. LDLR$^{22\text{-}788}$-Fc recognized as low as 10 pmol of LRPAP1 but failed to detect Tcnα on the NC membrane.

Supplementary information, Fig. S5

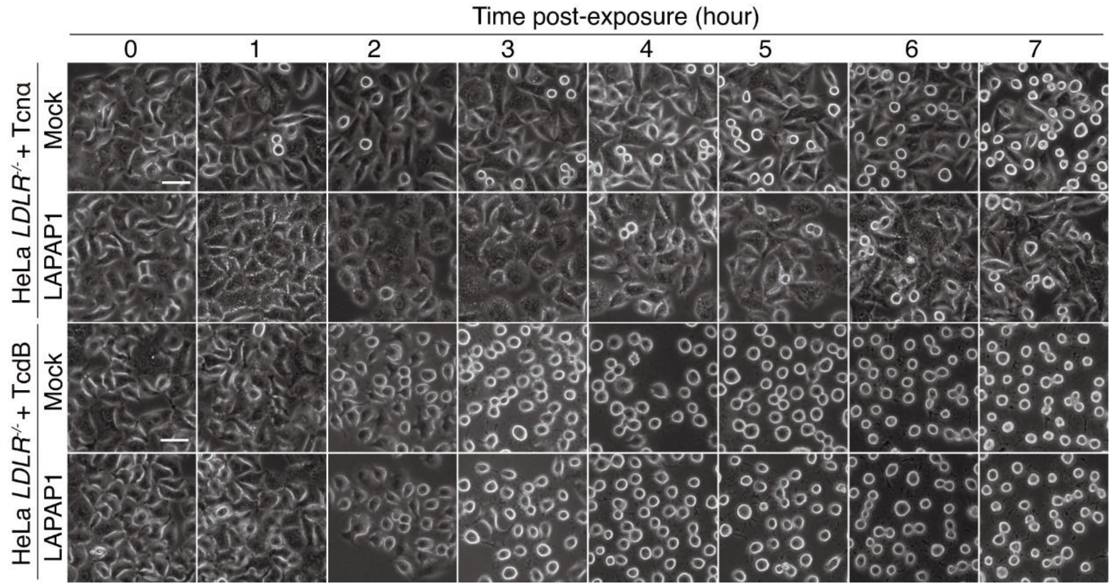

LRPAP1 further protected *LDLR*$^{-/-}$ cells from Tcnα. Representative images showing that LRPAP1 further protected HeLa *LDLR*$^{-/-}$ cells from Tcnα, but not from TcdB. (Scale bar represents 50 μm)

Supplementary information, Fig. S6

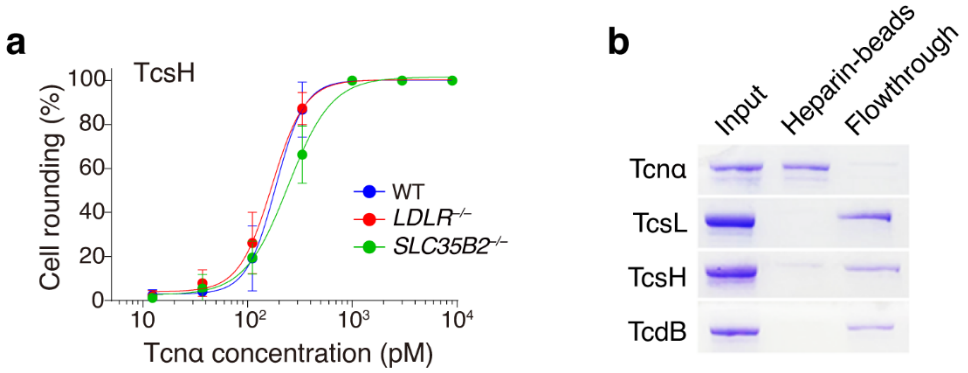

TcsH, TcsL, and TcdB do not recognize sulfated glycosaminoglycans. a Sensitivities of the HeLa WT, *LDLR*$^{-/-}$, and *SLC35B2*$^{-/-}$ cells to TcsH were measured using the cytopathic cell-rounding assay. The percentages of rounded cells were quantified, plotted, and fitted. **b** Tcnα, but not TcsH, TcsL, and TcdB, bound to the heparin-beads in the pulldown assay.

Supplementary information, Fig. S7

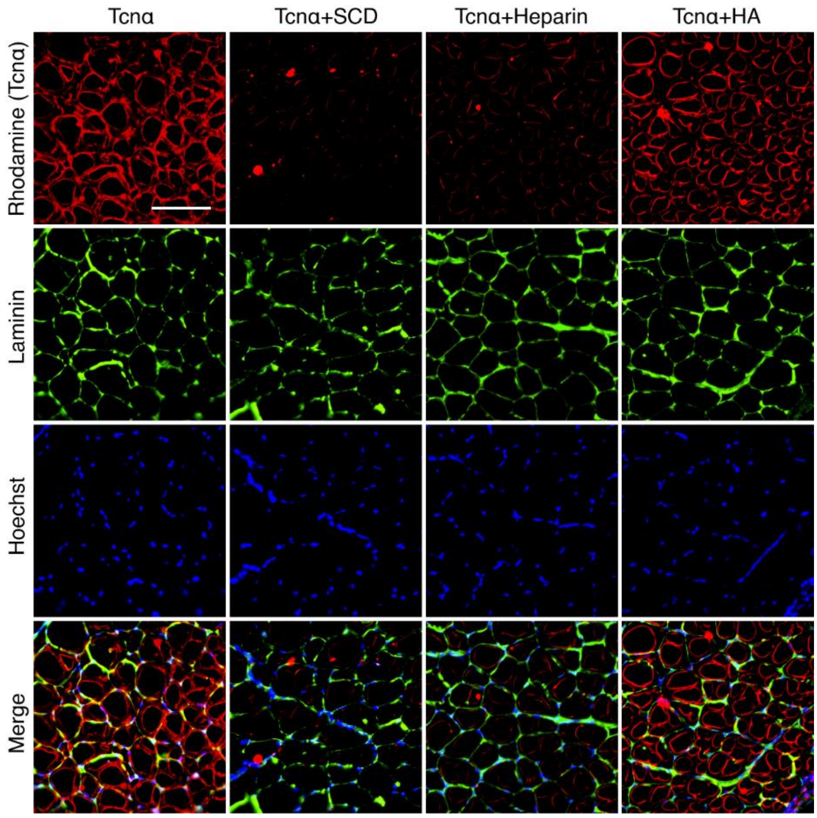

Sulfated α-cyclodextrin and heparin blocked binding of Tcnα to muscle sections. Representative confocal fluorescence images showing that pre-incubation of sulfated α-cyclodextrin (SCD) or heparin, but not hyaluronic acid (HA) (all at 1 mg/ml), blocked the binding of Rhodamine-labeled Tcnα (red) to the mouse tibialis anterior muscle sections. Laminin (green) was labeled to indicate the basement membrane of skeletal muscles. Cell nuclei (blue) were stained by Hoechst. (Scale bar represents 100 μm)

Supplementary information, Fig. S8

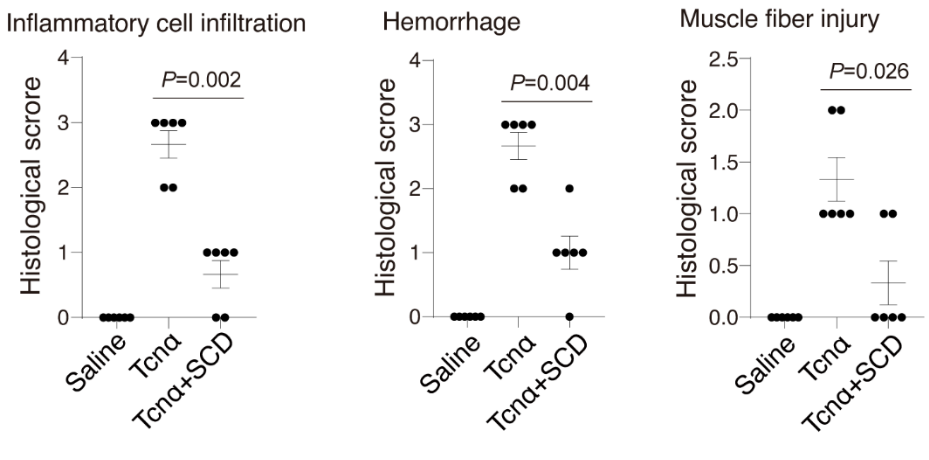

Sulfated α-cyclodextrin reduced Tcnα-induced myopathies.

Histopathological scores were accessed based on inflammatory cell infiltration, hemorrhage, and muscle fiber injury, respectively. (Data are mean ±s.e.m., Mann-Whitney test.

Materials and Methods

Cell lines, antibodies, and reagents.

HeLa (H1, CRL-1958) and 293T (CRL-3216) cells were originally obtained from ATCC. They tested negative for mycoplasma contamination and authenticated via STR profiling. Hela *LDLR*$^{-/-}$, *EXT2*$^{-/-}$, *EXTL3*$^{-/-}$, *SLC35B2*$^{-/-}$, *SLC35B2*$^{-/-}$/*LDLR*$^{-/-}$, and *CSPG4*$^{-/-}$ cells were laboratory stocks and generated as previously described[1, 2]. The following reagents and antibodies were purchased from the indicated vendors: Hoechst 33258 staining solution (BBI, E607301), anti-laminin rabbit antibody (Sigma, L9393), and Alexa Fluor 488 goat anti-rabbit IgG (Abcam, ab150077).

Genes and plasmids.

The DNA sequences encoding Tcnα (*Clostridium novyi* A strain GD211209) and TcdB (*Clostridioides difficile* strain 630) were codon-optimized, synthesized by Genscript (Nanjing, China), and cloned into a modified pHT01 vector with a His-tag introduced to their C-terminus. The expression plasmid pQTEV-LRPAP1 encoding LRPAP1 was obtained from Addgene (#31327). The DNA fragments encoding the ectodomain of human LDLR (LDLR$^{22\text{-}788}$) and IgG1 Fc were fused and cloned into the pHLsec vector as previously described[2]. The DNA fragments encoding mouse Ldlr were cloned into the plasmid pLVX-IRES-mCherry (Miaoling Bioscience & Technology Co., Ltd, P0424).

Protein purification.

Recombinant Tcnα and TcdB were expressed in *Bacillus subtilis* SL401 and purified as His6-tagged proteins as previously described[3]. In brief, *B. subtilis* cells were cultured at 37 °C till OD_{600} reached 0.6 and induced with 1 mM isopropyl-β-D-thiogalactoside (IPTG) at 25 °C for 20 hours. LRPAP1 was expressed in *Escherichia coli* BL21 (DE3) and purified as His_6-tagged proteins. *E. coli* cells were cultured at 37 °C till OD_{600} reached 0.6 and then induced with 0.25 mM IPTG at 18 °C for 16 hours. The recombinant proteins were purified by Ni-affinity chromatography and size-exclusion chromatography (GE Healthcare). Recombinant $LDLR^{22\text{-}788}$-Fc protein was expressed in HEK293F cells. HEK293F cells were cultured in SMM 293-T II serum-free medium (Sino Biological) supplemented with 100 μg/ml Penicillin-Streptomycin (Hyclone) at 37°C under 5% CO_2. Cells were transfected with Polyethylenimine Linear (Yeasen Biotech, MW40000) with a density of 2.0-3.0$\times 10^6$ cells per ml. After 5 days, the culture medium was collected, and the recombinant protein was purified by Ni-affinity chromatography and size-exclusion chromatography (GE Healthcare).

Genome-wide CRISPR/Cas9 screening with Tcnα.

HeLa CRISPR/Cas9 genome-wide KO library was generated as previously described[1]. The GeCKO v2 library is composed of two sub-libraries with each contains three unique sgRNA per gene and was independently prepared and screened[4]. HeLa-Cas9 cells were transduced with sgRNA lentiviral library at a multiplicity of infection of 0.3 and selected with 2.5 μg/ml puromycin. For each CRISPR sub-library, ~8×10^7 cells

were plated onto two 15-cm cell culture dishes to ensure sufficient sgRNA coverage. These cells were exposed to Tcnα for 16 hours and then washed with phosphate buffer saline (PBS) to remove loosely attached cells. The remaining cells were cultured with toxin-free medium to ~70% confluence and subjected to the next round of screening with higher concentrations of toxins. Three rounds of screenings were performed with increasing concentrations of Tcnα (160, 400, and 750 pM). The remaining cells were collected and their genomic DNA was extracted using the Blood and Cell Culture DNA mini kit (Qiagen). DNA fragments containing the sgRNA sequences were amplified by PCR using primers lentiGP1_F (AATGGACTATCATATGCTTACCGTAACTTGAAAGTATTTCG) and lentiGP-3_R (ATGAATACTGCCATTTGTCTCAAGATCTAGTTACGC). The next-generation sequencing was performed by the commercial vendor (Novogene, Beijing).

HeLa CRISPR/Cas9 KO cells.

To generate HeLa *B4GALT7*$^{-/-}$, *EXT1*$^{-/-}$, *EXT2*$^{-/-}$, *EXTL3*$^{-/-}$ cells, the following sgRNA sequences were cloned into LentiGuide-Puro vectors (Addgene #52963) to target the indicated genes: 5′-CAGCAGGATGCCGCCGACAT-3′ (B4GALT7), 5′-TCTCCTTCAATGACGACGAC-3′ (EXT1), 5′-CGATTACCCACAGGTGCTAC-3′ (EXT2), 5′-GAGGTGAGCATCGTCATCAA-3′ (EXTL3). Lentiviruses were generated by transfecting 293T cells with LentiGuide-Puro containing each sgRNA, pSPAX2, and pMD2g. HeLa-Cas9 cells were transduced with lentiviruses that express the sgRNAs. Mixed populations of infected cells were selected with puromycin (2.5

μg/ml). For all mixed populations of KO cells, NGS analysis was performed and all mixed cells showed over 60% of the KO efficiency.

Cytopathic cell-rounding experiment.

The cytopathic effect of Tcnα and TcdB was analyzed using the standard cell-rounding assay. In brief, cells were exposed to Tcnα and TcdB for 10 hours, and phase-contrast images of cells were recorded (Olympus IX73; ×10 or ×20 objectives). A zone of 300×300 μm was selected randomly, containing 50-150 cells. The numbers of normal and round-shaped cells were counted manually. The percentage of round-shaped cells was analyzed using the GraphPad Prism software (ver. 9.0.0, GraphPad Software, LLC).

Competition assays with glycans, surfen, or LRPAP1.

For competition with glycans, Tcnα (7.5 nM) was pre-mixed with or without 1mg/ml heparan sulfate (Macklin, H876459), chondroitin sulfate (Sigma, C9819), dextran sulfate (Sigma, D4911), hyaluronic acid (Sigma, 53747), heparin (J&K, A16198), sulfated α-cyclodextrin (Sigma-Aldrich, 494542-5G) in fresh DMEM medium and incubated at 37 °C for 20 min. The mixture was then added to the cells. Cells were incubated at 37 °C and the percentage of rounded cells over time was recorded and analyzed. For competition with surfen, cells were pre-mixed with or without surfen hydrate (Sigma-Aldrich, S6951) of indicated concentrations at 37 °C for 30 minutes. For competition with LRPAP1, cells were pre-incubated with LRPAP1 protein (1000-fold in molar versus the toxin) or in the medium at 37 °C for 20 min. The medium was

then supplemented with 10 nM Tcnα or 2.5 pM TcdB. Cells were incubated further at 37 °C and the percentage of rounded cells over time was recorded and analyzed.

Surface binding of Tcnα and TcdB on HeLa cells.

Tcnα and TcdB were labeled using an NHS-Rhodamine fluorescent labeling kit (#46406, ThermoFisher Scientific) following the manufacturer's instruction. Hela WT, *CSPG4*$^{-/-}$, *LDLR*$^{-/-}$, and *SLC35B2*$^{-/-}$ cells were incubated with 100 nM Rhodamine-labeled Tcnα or 25 nM Rhodamine-labeled TcdB in PBS for 30 min on ice. Cells were washed five times with ice-cold PBS and fixed with 4% paraformaldehyde (PFA) and the cell nuclei were labeled with Hoechst. Confocal images were captured with the Zeiss LSM 880 NLO with AiryScan System.

Binding of Tcnα on mouse muscle tissue sections.

Mice tibial anterior muscles were dissected out, embedded with optimal cutting temperature compound (Leica Biosystems), frozen, and cut into 10 μm thick sections with a cryostat (CM1950, Leica Biosystems). The tissue sections were incubated with Rhodamine-labeled Tcnα (25 nM) without or with heparin, sulfated α-cyclodextrin, and hyaluronic acid (5 mg/ml) at room temperature for 30 min. The sections were then washed with PBS, fixed with 4% PFA, and blocked with 2% BSA plus 10% donkey serum. The sections were then incubated with an anti-laminin rabbit antibody (primary antibody, 1:400) followed by the incubation with Alexa Fluor 488 goat anti-rabbit (secondary antibody, 1:500) and Hoechst dye. Confocal images were captured with the

Nikon A1 confocal system.

Tcnα tibialis anterior muscle injection assay and histological analysis.

BALB/c mice (6-8 weeks, female, specific-pathogen-free) were purchase from the Laboratory Animal Resources Center at Westlake University (Hangzhou, China). Mice were housed under the specific-pathogen-free condition with free access to drinking water and food during the experiments. Mice were anesthetized with pentobarbital sodium. Twenty microliters of Tcnα (100 ng in saline), Tcnα premixed with sulfated α-cyclodextrin (100 ng of Tcnα plus 2 mg of sulfated α-cyclodextrin in saline), or saline alone was injected into each anterior tibial muscle of the mouse. Mice were recovered under a 37 °C condition. After four hours, all mice were euthanized with CO_2 gas and tibial anterior muscles were extracted. Specimens were fixed in formalin for 12 hours and dehydrated with alcohol, cleared by xylene, and embedded in paraffin. Paraffin blocks were cut into 4 μm thick sections and subjected to H&E staining. The pathology in muscles was scored blindly based on hemorrhage, inflammatory cell infiltration, and muscle fiber injury on a scale of 0–3 (normal, mild, moderate, or severe).

Ethics statement.

The intramuscular injection of Tcnα in mice was performed following the institutional guidelines. All animal procedures reported herein were approved by the Institutional Animal Care and Use Committee at Westlake University (IACUC Protocol #20-046-TL). To minimize the distress and pain, the mice were monitored every hour. Animals

with signs of pain or distress such as labored breathing, inability to move after gentle stimulation, or disorientation were euthanized immediately. This method was approved by the IACUC and monitored with a qualified veterinarian.

Statistical analysis.

Statistical analysis was performed using GraphPad Prism software (ver. 9.0.0, GraphPad Software, LLC) and SPSS (ver. 22, IBM) software. Experimental groups were compared using the two-sided Mann-Whitney test (Fig. 1d, f, i, l, n, and r; Supplementary information, Fig. S7). All data were obtained from independent experiments.

Data availability.

All data are fully available without restriction.

References

1 Tao L, Zhang J, Meraner P *et al.* Frizzled proteins are colonic epithelial receptors for C. difficile toxin B. *Nature* 2016; **538**:350-355.

2 Tao L, Tian S, Zhang J *et al.* Sulfated glycosaminoglycans and low-density lipoprotein receptor contribute to Clostridium difficile toxin A entry into cells. *Nat Microbiol* 2019.

3 Shen E, Zhu K, Li D *et al.* Subtyping analysis reveals new variants and accelerated evolution of Clostridioides difficile toxin B. *Communications Biology* 2020; **3**:347.

4 Sanjana NE, Shalem O, Zhang F. Improved vectors and genome-wide libraries for CRISPR screening. *Nat Methods* 2014; **11**:783-784.
